# The Role of Scanning Electron Microscopy in the Direct Diagnosis of Onychomycosis

**DOI:** 10.1155/2018/1581495

**Published:** 2018-01-09

**Authors:** Xueping Yue, Aiping Wang, Qing Li

**Affiliations:** ^1^Department of Dermatology, Beijing Tian Tan Hospital, Capital Medical University, Beijing 100050, China; ^2^Department of Dermatology & Venereology, Peking University First Hospital and Research Center for Medical Mycology, Peking University, Beijing 100034, China

## Abstract

**Purpose:**

The purpose of this study was to evaluate the role of scanning electron microscopy (SEM) in the direct diagnosis of suspected onychomycosis with negative mycological test results.

**Methods:**

Outpatients diagnosed with suspected onychomycosis with negative mycological test results, including direct microscopic examination with 10% potassium hydroxide (KOH) and fungal culture, on 3 separate occasions were recruited. A small piece of infected nail was obtained for SEM examination.

**Results:**

Among the 48 suspected onychomycosis samples, SEM revealed that 18 (37.5%) were positive for fungal structures, including 10 (20.8%) cases of hyphae and 8 (16.7%) cases of yeast blastospores or budding.

**Conclusion:**

SEM represents an effective method to diagnose suspected onychomycosis when the traditional mycological methods were negative. Therefore, this technique could be used in clinical practice.

## 1. Introduction

Onychomycosis is a common fungal infection in dermatologic clinics that is caused by dermatophyte,* Candida*, or nondermatophyte mold. The frequently used mycological tests include direct microscopic examination with 10% potassium hydroxide (KOH) and fungal culture. However, the positive rate of these techniques is not high. Occasionally, although an infected nail is highly suspected in the clinic, the mycological tests are consistently negative. In this situation, patients are advised to undergo regular follow-up or antifungal experimental treatment. Therefore, the accurate diagnosis of fungal infection is important for clinical practice. Thus, we aimed to identify a new method to increase the diagnostic positive rate of onychomycosis.

Other diagnostic methods include histopathological examination of periodic acid-Schiff (PAS) staining of nail clippings or damaged nail plates [1, 2] or polymerase chain reaction (PCR) [3, 4]. Although these techniques have a high positive rate, the nail biopsy procedure is complicated and time-consuming. In addition, PCR can only be used in several prominent fungal laboratories. No commercial fungal PCR reagent is available in China, which limits the application of PCR in a clinical setting.

In clinical practice, scanning electron microscopy (SEM) has been commonly used for observation of the ultrastructure of numerous materials, pathogens, and diseases. SEM images can be magnified thousands of times. In the mycological field, several studies have demonstrated that SEM is a good method for observing fungal ultrastructure in nails with onychomycosis [5–9]. Observation of a dermatophyte by SEM [5–9] reveals hyphae with clearly visible branches and septa or arthroconidia, whereas analysis of* Candida* in culture medium by SEM [10, 11] often shows pseudohyphae, blastospores, budding, and a ring of bud scars. Thus, SEM is a good tool for observing fungal morphology. However, this technique has seldom been used for the diagnosis of fungal infection. Jian et al. [8] studied 48 cases of suspected onychomycosis using SEM and demonstrated that the positive rates by SEM, histopathology, fungal culture, and KOH were 87.5%, 60.42%, 41.67%, and 27.08%, respectively. In our previous study [12, 13], we used SEM to observe the* in vivo* ultrastructural characteristics of fungi or* Trichophyton rubrum* in onychomycosis. In addition, SEM was also used to improve therapy for onychomycosis with negative fungal cultures. Both of these studies demonstrated that SEM was a simple, clear tool to observe the fungal structure in the infected nail* in vivo*, and a relatively large sample was easily obtained for SEM. Therefore, in this study, SEM was applied in the cases of suspected onychomycosis with negative mycological tests to increase the diagnostic positive rate of onychomycosis and provide better therapeutic strategies.

## 2. Materials and Methods

### 2.1. Clinical Data

Inclusion criteria were as follows: outpatients diagnosed with suspected onychomycosis with negative 10% KOH direct microscopic examination and fungal cultures on 3 separate occasions were recruited from October 2014 to May 2016 in the department of dermatology clinic of Beijing Tian Tan Hospital, Capital Medical University. No restrictions on age, gender and antifungal therapy were imposed. The following exclusion criteria were employed: any nail lesions that could be definitely diagnosed as onychomycosis, onycholysis, twenty-nail dystrophy, subungual hemorrhage, or nail disease caused by lichen planus or psoriasis. This study was approved by the Beijing Tian Tan Hospital, Capital Medical University ethics committee. Study subjects were informed and signed a written consent form.

### 2.2. 10% KOH Examination and Fungal Culture

Mycological analysis, including KOH examination and fungal culture, was performed as described in the author's previous study [13]. First, some debris from an infected nail was applied on a slide with a drop of 10% KOH and observed by direct microscopy. Then, additional debris was inoculated on Sabouraud Dextrose Agar (SDA) and incubated at 28°C for 1–4 weeks. All the patients were examined on 3 separate occasions. Samples that showed negative results on all 3 occasions were prepared for SEM examination.

### 2.3. SEM Preparation and Observation of Nail Samples

The samples were prepared in accordance with the authors' previous studies [10–12]. The infected nail plate with the width greater than 4 mm was removed and fixed. Then, the sample was washed, postfixed, dehydrated, displaced, dried, sprayed, and chemically dried. Hitachi TM-1000 (Hitachi, Japan) SEM was used to observe and photograph the samples.

## 3. Results

### 3.1. Basic Clinical Information and SEM Results

In total, 48 patients with suspected onychomycosis were recruited, including 48 infected nails. The clinical characteristics included color change (yellow or gray-black), nail plate thickening, subungual debris accumulation, and damaged nail plates. The samples showed negative KOH and fungal culture results on 3 separate occasions. However, out of the 48 samples, 18 (37.5%) were positively diagnosed by SEM, with detection of 10 (20.8%) cases of hyphae and 8 (16.7%) cases of yeast blastospores or budding.

### 3.2. Fungal Ultrastructure Based on SEM Observation

**Figure 1:**
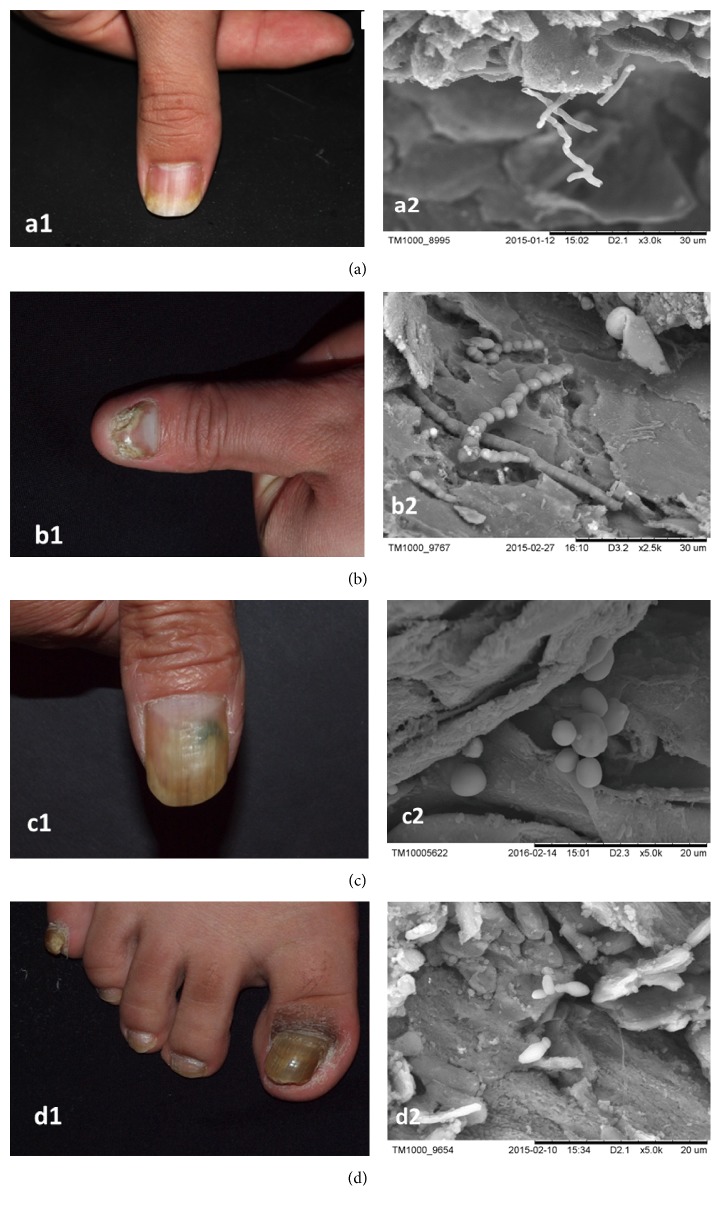
(a1) The yellowish thumb of case 1 with nail separation. (a2) SEM observation of case 1. The hyphae are smooth, intact, curved, and mellow with branches or local dryness (×3000). (b1) The yellowish thumb of case 2 with partial nail plate absence. (b2™) SEM observation of case 2. The clear arthroconidia are showed (×2500). (c1) The yellowish thumb of case 3 with a partial blackish nail. (c2) SEM observation of case 3. The yeast blastospores, with the ring of bud scars, exhibit local accumulation (×3000). (d1) The yellowish thumb of case 4 with nail plate thickening. (d2) SEM observation of case 4. Dispersed blastospores with budding, some of which resemble a bowling pin (×3000).

Some of the 48 nail plates were damaged with disorganized layers. Hyphae appeared in 10 samples (Figures 1(a) and 1(b), cases 1 and 2), similar to dermatophyte morphology. Some hyphae appeared smooth, intact, curved, and mellow with branches or local dryness, whereas some were arthroconidia (Figure 1(b2)). Yeast blastospores, budding, and a ring of bud scars were identified in 8 samples (Figures 1(c) and 1(d), cases 3 and 4), which indicating yeast morphology. The spores were dispersed or locally accumulated. The structure of some budding yeast resembled a bowling pin (Figure 1(d2)), and no typical pseudohyphae were observed.

## 4. Discussion

Although the traditional mycological methods using KOH and fungal culture are easy to perform, their relatively low positive rates are a big problem in clinical practice. Therefore, developing and testing a new diagnostic method is necessary. However, SEM is seldom used for the diagnosis of onychomycosis. In this study, 48 suspected onychomycosis cases showed negative KOH and fungal culture results on 3 separate occasions; however, 37.5% (18 samples) of the samples exhibited hyphae and yeast blastospores on SEM. These results demonstrate that SEM is an effective method for diagnosing onychomycosis when traditional mycological examinations have consistent negative results. This method represents a novel application of SEM in dermatology. Moreover, the morphologies of hyphae and yeast blastospores in our study are similar to those reported in other studies [8–13]. Therefore, this method could be used to preliminarily estimate the strain and aid in the selection of a sensitive antifungal drug. This is another advantage of this research, as reported in the author's previous study [12].

Jian et al. [8] studied 48 suspected onychomycosis cases using SEM and demonstrated that the positive rate of SEM was 87.5%. In contrast with their study, we only recruited cases of suspected onychomycosis with negative mycological examination results, which explains why our positive rate was lower. Moreover, more than one-third of these samples were positive for onychomycosis as assessed by SEM; therefore, more effective and accurate methods are needed to increase the diagnostic positive rate and to avoid misdiagnosis when traditional mycological examination results are negative. SEM is a very effective, accurate diagnostic method, and samples are easy to attain for SEM analysis.

In our study, arthroconidia were clearly identified via SEM. This morphology was also observed by KOH direct microscopy. It was deduced that this morphology may be self-protective when the host's immune system was more active or after antifungal treatment. This morphology was clearly demonstrated first by SEM, which has never been previously reported before. The technique was useful for obtaining more information about fungal morphology* in vivo*.

However, SEM still has some limitations. For example, 2-3 days are needed to prepare samples. This method is expensive and is not normally available at all hospitals. However, some samples could be fixed and then sent to another laboratory that performs SEM.

In conclusion, we suggest that SEM is a simple, accurate, and effective method to identify onychomycosis when traditional mycological tests are negative. SEM also provides better information for the selection of an optimal antifungal drug. Therefore, it could be applied in clinical practice.
